# Optimal timing for intubation in patients on non‐invasive ventilation: A retrospective cohort study

**DOI:** 10.1002/hsr2.1757

**Published:** 2023-12-11

**Authors:** Tatsuhiko Abe, Toshishige Takagi, Kazunari Takahashi, Kosuke Yagi, Ai Tsuge, Tomoko Fujii

**Affiliations:** ^1^ Department of Intensive Care Jikei University Hospital Tokyo Japan; ^2^ Department of Clinical Engineering Technology Jikei University Hospital Tokyo Japan

**Keywords:** intensive care units, intubation, non‐invasive ventilation, respiratory insufficiency

## Abstract

**Background and Aims:**

The timing of transition from non‐invasive ventilation (NIV) to invasive ventilation in the intensive care unit (ICU) is uncertain due to a lack of clinical evidence. This study aimed to identify the optimal timing of intubation in patients with respiratory failure managed with NIVs.

**Methods:**

A single‐center observational study was conducted in Tokyo, Japan. Patients in the ICU managed with NIV between 2013 and 2022 were screened. The primary outcome was 28‐day invasive ventilator‐free days. Statistical analyses used locally estimated scatter plot smoothing (LOESS) and generalized linear mixed models to estimate the association between the timing of transition and prolonged intubation duration.

**Results:**

During the study period, 139 of 589 adult ICU patients receiving NIV transitioned to invasive ventilation. The LOESS curve indicated the longest 28‐day ventilator‐free days around 24 h after NIV initiation, after which the primary outcome decreased linearly. Late intubation after 24 h of NIV initiation was associated with fewer 28‐day ventilator‐free days (adjusted mean difference: −0.22 days [95% confidence interval: −0.31, −0.13]).

**Conclusion:**

We identified a non‐linear association between the timing of intubation and 28‐day invasive ventilator‐free days. The critical 24‐h time window for patients on NIV was associated with longer 28‐day invasive ventilator‐free days.

## Key points

- The timing of transition from non‐invasive ventilation (NIV) to invasive ventilation in the ICU is uncertain.
- This study showed that the 28‐day invasive ventilator‐free days peaked around 24 h after the start of NIV for patients with respiratory failure who required tracheal intubation after using NIV.
- Patients using NIV should be closely monitored and evaluated within 24‐h window to determine if tracheal intubation is necessary.

## 1 INTRODUCTION

Non‐invasive ventilation (NIV) including non‐invasive positive pressure ventilation (NPPV) and high flow nasal cannula (HFNC) have been in clinical use for over 50 years and are now essential equipment in the intensive care unit (ICU) worldwide.^1^ Previous studies have shown that NIV reduces the need for invasive ventilatory support and decreases mortality compared to conventional oxygen therapy.^1^, ^2^, ^3^, ^4^, ^5^ However, many patients still require invasive ventilatory support even when using NIV. The timing of transition to invasive ventilation is critical, as delayed tracheal intubation in patients requiring invasive ventilation can worsen their prognosis; however, there are no clear criteria for when to transition to invasive ventilation.^6^

A previous study analyzed data from patients who started using HFNC but later required tracheal intubation. They reported that patients intubated after 48 h had a higher mortality rate than those intubated within 48 h.^7^ Similar trends were observed for successful extubation, ventilator‐weaning and ventilator‐free days up to the 28th day. Despite advances in NIV have enabled the management of acute respiratory distress syndrome (ARDS) without invasive ventilation, the effectiveness of this approach is limited. Notably, about a third of ARDS patients required intubation within 3 days with higher mortality than those who were intubated early.^8^

Currently, international guidelines of NIV provided by ERS/ATS in 2017 do not specify when to switch to tracheal intubation.^5^ In contrast, a guideline of NIV by British Thoracic Society recommends assessing a patient's respiratory status 4–6 h after initiating NIV to determine whether to transition to invasive ventilation.^9^ Nonetheless, the lack of clinical data that provide sufficient evidence to recommend 4–6 h in the guideline underscores the uncertainty in identifying the optimal timing for the transition.

Therefore, we conducted an observational study to examine the optimal timing for patients under NIV management to decide when to switch to endotracheal intubation, with invasive ventilator‐free days at 28 days as the primary outcome. Determining the timeline could improve patient outcomes and help critical care professionals make well‐informed decisions in patient management in the ICU.

## 2 METHODS

We conducted a single‐center, observational study in an academic hospital in Tokyo, Japan, which has 1075 beds for acute care admission and 20 beds in the general ICU. We screened all patients admitted to the ICU between April 1, 2013, and March 31, 2022, using electronic health records and ICU patient database.

We included all patients who were treated with NPPV or HFNC and subsequently transitioned to invasive ventilatory support due to insufficient response to NPPV or HFNC treatment. We excluded patients under 18 years of age, those who received NIV to prevent reintubation after extubation, and those who did not provide consent.

We collected data from electronic medical records and a local ICU database including age, gender, height, weight, chronic disease (heart failure, respiratory failure, liver failure, cancer metastasis, immunosuppression, chronic dialysis), ICU admission route, emergency or scheduled admission, and acute physiology and chronic health evaluation (APACHE) II score. Arterial blood gas analysis, vital signs (systolic blood pressure, diastolic blood pressure, mean blood pressure, heart rate, respiratory rate and percutaneous oxygen saturation), and ROX index (SpO2/FiO2/respiratory rate)^10^ were collected at two points: just before NIV and invasive ventilatory management.

The primary outcome was invasive ventilator‐free days at 28 day, defined as the time a patient was alive without invasive ventilation within 28 days after initiating invasive ventilator management, which is a critically important outcome for patients with acute respiratory failure. This outcome was chosen because the outcome assesses the impact on ventilator dependency taking the competing risk of death into account. If patients died within 28 days post‐intubation, the invasive ventilator‐free days at 28‐day were recorded as zero. Assigning zero to those patients ensure that exposures that decrease ventilator time but increase mortality are not mistakenly interpreted as beneficial. If a patient was weaned from the ventilator after tracheostomy, the post‐weaning period was recorded. Secondary outcomes were ICU mortality, hospital mortality, length of ICU stay, length of hospital stay, and tracheostomy.

### 2.1 Statistical methods

Patient characteristics and outcome summary statistics were described. Continuous data were presented as medians with interquartile range (IQR), while count data were presented as absolute numbers with percentages. Locally estimated scatter plot smoothing (LOESS) techniques were employed to examine the possible non‐linear associations between ventilator‐free days at 28‐day and the number of days required to switch from NIV initiation to tracheal intubation. The smoothed curve was utilized to visually inspect the optimal timing of transition from NIV to invasive ventilation. Patient who died within 24 h of ICU admission was excluded from the plot as the observation period was too short to assess the clinical course and those patients were deemed to die despite all the effort.

The primary analysis aimed to evaluate the association of possible risk factors for prolonged duration of intubation, which cut‐off was identified from the LOESS curve, with the outcomes using generalized linear mixed models (GLMM). GLMM was used to take into account the potential heterogeneity of the association derived from the difference of NPPV and HFNC. Normality test was not performed as was not applicable for the response variable in the model. The model covariates included the change in ROX index from the start of NIV to intubation, APACHE II score, age, gender, and route of ICU admission as prognostic factors requiring adjustment. These variables were selected based on clinical relevance and findings from previous studies.^10^ We investigated all patients with NIV who did not respond to treatment and required intubation, including patients with COPD and acute heart failure with pulmonary congestion. Given those patients with COPD or acute pulmonary edema are rarely intubated, and only a small number of patients were included in the study population (seven in total), those characteristics were not selected for the adjustment.

We performed sensitivity analyses to ensure the robustness of the primary analysis for the primary outcome. In these sensitivity analyses, the change in ROX index in the model was replaced with changes in respiratory rate and PaO2/FiO2, or those variables at the start of NIV or at intubation. The additional analysis was performed to explore the possible bias due to the exclusion of early death. There were no missing data in the study dataset. All analyses were performed using R version 4.2.2 (R Foundation for Statistical Computing, Vienna, Austria). A two‐tailed *p* < 0.05 was considered to be statistically significant.

### 2.2 Ethics

The study protocol was reviewed and approved by the Jikei University Ethics Committee (34‐107(11254)), and the need for informed consent was waived due to its retrospective observational design that involved no intervention and no personal information. All study processes were carried out in accordance with relevant guidelines and regulations.

## 3 RESULTS

During the study period, 589 adult ICU patients were treated with NPPV or HFNC. Of these, 148 patients transitioned to invasive ventilation. Nine patients were excluded (six for pre‐operative management and three for intubation after being weaned off from NIV), so that 139 patients were eligible (Figure S1).

Patient characteristics are presented in Table 1. The median patient age was 70 years (IQR: [58, 78]), body mass index was 21.4 [19.5, 25.7], and APACHE II score was 25 [20, 30]. The route of ICU admission was mainly the ward (74.8%), followed by the emergency room (20.9%), and other sources (4.4%). For chronic diseases, immunosuppression was the most common (30.9%), followed by chronic dialysis (6.5%), respiratory failure (4.3%), cancer metastasis (3.6%), liver failure (2.2%), and heart failure (0.7%).

**Table 1:** Overall patient characteristics at ICU admission (*N* = 139).

Characteristics	Median [IQR] or *n* (%)
Age	70 [58, 78]
Sex, male	100 (71.9)
BMI, kg/m^2^	21.4 [19.5, 25.7]
Emergency admission	138 (99.3)
Route	
Ward	104 (74.8)
Emergency room	29 (20.9)
Other	6 (4.4)
Chronic conditions	
Immunosuppression	43 (30.9)
Dialysis	9 (6.5)
Respiratory failure	6 (4.3)
Cancer metastasis	5 (3.6)
Liver failure	3 (2.2)
Heart failure	1 (0.7)
Glasgow Coma Scale	15 [14, 15]
APACHE II score	25 [20, 30]
COVID‐19 pneumonia	22 (15.8)

Abbreviations: APACHE, acute physiology and chronic health evaluation; BMI, body mass index; IQR, interquartile range.

Vital signs and arterial blood gas analyses at initiating NIV and transition to intubation are shown in Table 2. Most patients presented with type I acute respiratory failure mainly caused by pneumonia at the beginning. The rapid respiratory rate at NIV initiation was attenuated at the timing of intubation, and ROX index increased. However, PaCO2 increased, and arterial pH was decreased by the timing of intubation.

**Table 2:** Vital signs and arterial blood gas analyses at the start of NIV and invasive mechanical ventilation.

	Pre‐NIV	Pre‐intubation
Respiratory rate	27 [21, 33]	20 [15, 25]
SpO2, %	94 [92, 97]	95 [90, 98]
ROX index	5.05 [3.79, 8.08]	6.27 [4.36, 8.17]
PaO2/FiO2	126 [91, 181]	126 [84, 186]
Heart rate, bpm	102 [87, 117]	103 [85, 123]
Mean arterial pressure, mmHg	86 [74, 98]	83 [67, 98]
FiO2, %	70 [50, 80]	80 [60, 100]
pH	7.42 [7.35, 7.47]	7.35 [7.25, 7.43]
PaO2, mmHg	77.6 [67.0, 101.5]	86.4 [70.1, 123]
PaCO2, mmHg	34.7 [29.8, 43.4]	45.9 [35.6, 55.6]
HCO_3_^−^, mmol/L	22.8 [19.9, 26.5]	23.3 [20.6, 27.3]
Base excess, mmol/L	−0.5 [−3.7, 2.2]	−1.2 [−5.4, 2.3]
Lactate, mmol/L	1.3 [1.0, 2.0]	1.3 [1.1, 2.3]

*Note*: ROX index was calculated as SpO2/FiO2/respiratory rate.

Abbreviation: NIV, non‐invasive ventilation.

The median time to intubation from starting NIV was 0.59 [0.12, 1.62] days. In the overall study population, the median ventilator‐free days at 28‐day was 18.1 [0.0, 24.2], and the other clinical outcomes are presented in Table 3.

**Table 3:** Clinical outcomes.

Outcomes	Median [IQR] or *n* (%)
Ventilator‐free days at day 28, days	18.1 [0.0, 24.3]
ICU mortality	41 (29.5)
Hospital mortality	60 (43.2)
Length of ICU stay, days	9.9 [5.0, 16.8]
Length of hospital stay, days	45 [23, 99]
Tracheostomy	26 (18.7)

Abbreviation: ICU, intensive care unit.

**Figure 1:**
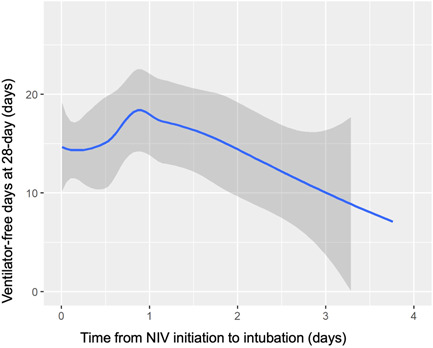
The relationship between invasive ventilator‐free days at 28‐day and the number of days from NIV initiation to tracheal intubation. NIV, non‐invasive ventilation.

The non‐linear curve with LOESS technique presenting the association between time from NIV to intubation and invasive ventilator‐free days at 28‐day showed a peak around 24 h after the start of NIV (Figure 1). After 24 h, the 28‐day invasive ventilator‐free days decreased linearly. Then time from NIV to intubation was dichotomized at 24 h and was explored for the association with the clinical outcomes. We used a GLMM to find that longer time from NIV to intubation (>24 h) was associated with shorter 28‐day invasive ventilator‐free days (Table 4).

**Table 4:** Association between late intubation and clinical outcomes.

Outcome	Adjusted mean difference or odds ratio	95% Confidence interval	*p* value
28‐day ventilator free days	−0.22	[−0.31, −0.13]	<0.001
ICU mortality	1.43	[0.56, 3.66]	0.46
Hospital mortality	3.06	[1.25, 7.51]	0.02
Length of ICU stay	0.27	[0.17, 0.37]	0.02
Length of hospital stay	0.12	[0.08, 0.16]	0.08
Tracheostomy	2.07	[0.79, 5.42]	0.14

Abbreviation: ICU, intensive care unit.

For secondary outcomes, longer time from NIV to intubation (>24 h) was associated with longer ICU stay, and higher hospital mortality (Table 4). There was no clear impact on hospital length of stay, ICU mortality, or the need for tracheostomy.

### 3.1 Sensitivity analyses

In all the sensitivity analyses, longer time from NIV to intubation (>24 h) was associated with shorter 28‐day invasive ventilator‐free days (Supporting Information Table). The additional analysis of non‐linear association including early death confirmed the findings in the main analysis (Figure S2).

## 4 DISCUSSION

### 4.1 Summary of key findings

In this study, we found a non‐linear relationship between the time from NIV to intubation and 28‐day invasive ventilator‐free days for patients with respiratory failure who required tracheal intubation after using NIV. The 28‐day invasive ventilator‐free days peaked around 24 h after the start of NIV, and the patients experienced longer ventilator‐free days if intubated within 24 h after NIV initiation. Our findings suggest that transition to intubation should be decided no later than 24 h of NIV treatment.

### 4.2 Context with prior literature

The NIV guideline by British Thoracic Society^9^ recommend assessing the effectiveness of NIV after 4–6 h to identify patients who need immediate intubation after starting NIV. Although the rationale for this was not provided, a study^11^ followed the guideline that mortality increased when patients were intubated more than 6 h after starting NIV. In major RCTs comparing the effects of NIV,^2^, ^3^, ^12^, ^13^, ^14^, ^15^, ^16^ the NIV period before intubation is typically within 48 h.

The non‐linear curve in the present study revealed that patients requiring intubation within the very early phase of the first 24 h of NIV carried a poor prognosis, possibly including those unsuitable for NIV treatment who were excluded in clinical trials of NIV. Our study provided insights into determining the intubation within 24 h in patients who appear to tolerate the initial phase but ultimately require invasive ventilation.

### 4.3 Implications for clinicians and future research

Delayed tracheal intubation is known to worsen prognosis in patients who need it. While Kang et al.^7^ reported that tracheal intubation after 48 h from the start of NIV negatively impacts prognosis, our study suggests that deciding to intubate around 24 h after NIV could be more beneficial. This difference may arise from the fact that long‐term NIV management, preserving spontaneous breathing, could contribute to patient self‐inflicted lung injury (p‐SILI) due to uncontrolled tidal volume,^17^, ^18^ resulting in adverse effects.

The multivariable analyses in this study demonstrated that ventilator‐free days at 28‐day were shorter for patients intubated more than 24 h after the start of NIV despite the improvement in ROX index. The ROX index is used to assist in assessing the effectiveness of NIV and predict the need for intubation. Our findings suggest that long observation period expecting for improved oxygenation result in a delayed tracheal intubation and a poor prognosis.

Patients' clinical course from the start of NIV to intubation showed that patients were transitioned to invasive ventilation when vital signs, oxygenation and metabolic derangement did not improve. Improvement in respiratory rate and ROX index (essentially respiratory rate in the formula) were observed, which could be attributed to sedation used to ease patients' dyspnea or agitation while applying NIV. In this study, the indication for intubation was left to the discretion of the attending clinicians. The cut‐off time of 24 h would need further validation through prospective documentation of indications for intubation to elaborate the impact of the timing. Clinically, making the decision to intubate shortly after the start of NIV is often straightforward, but determining the need for intubation beyond the early phase can be challenging.

There are limited studies addressing the optimal timing of intubation, and a clear consensus has yet to be established. Therefore, the results of the present study suggest that the first 24 h after initiation of NIV could be incorporated in the decision making of intubation.

### 4.4 Limitations

This study has several limitations. First, it is a single‐center study, which results could be influenced by local clinical practice and limits the generalizability of the results. However, the decision to intubate was made at the discretion of the attending intensive care physicians, who considers various factors, such as respiratory status, level of consciousness, and blood pressure, in line with clinical practice.

Second, the study did not address the mode of NPPV, nor did it evaluate detailed physiological data such as tidal volume or minute ventilation rate. Consequently, we cannot determine the extent to which preservation of spontaneous breathing may have induced lung injury. When using HFNC, caution is required because monitoring capabilities are limited, potentially triggering p‐SILI.

Third, the study did not evaluate the use of sedatives during NIV. Although the study site uses minimal sedatives when in need, the use of sedatives might have affected the assessment of vital signs such as respiratory rate and further clinical courses. Fourth, the study included patients with immunocompromised (30.9%) and COVID‐19 (15.8%). While specific subgroups might benefit from longer/shorter time to intubation, such enrichment was not feasible with the study population given the limited number of patients in each subgroup. For immunosuppressed patients, who should have greater disadvantages of tracheal intubation, such as the development of ventilator associated pneumonia, tailored protocol to apply NIV may work. Similarly, in patients with COVID‐19, pathophysiology and clinical course, i.e. responsiveness to NIV management, may differ from those of non‐COVID‐19 ARDS. Furthermore, the practice might vary depending on local availability of medical resources, including the infrastructure, equipment, and professional staff during the evolving phases of pandemic. Lastly, the timing of intubation needs further investigation in a study designed to control other confounding factors unmeasured in this study, ideally in a randomized trial to compare it with current practice. In this regard, this study provides a potential target threshold for future investigations.

## 5 CONCLUSIONS

In this observational study, time to intubation and ventilator‐free days had non‐linear association. Patients who used NIV and required tracheal intubation subsequently had longer invasive ventilator‐free days at 28‐day if they were managed with invasive ventilation around 24 h after NIV initiation. These findings suggest that patients using NIV should be closely monitored and evaluated within 24‐h window to determine if tracheal intubation is necessary.

## AUTHOR CONTRIBUTIONS

**Tatsuhiko Abe**: Conceptualization; Formal analysis; Methodology; Writing—original draft. **Toshishige Takagi**: Data curation; Writing—review & editing. **Kazunari Takahashi**: Data curation; Writing—review & editing. **Kosuke Yagi**: Data curation; Writing—review & editing. **Ai Tsuge**: Data curation; Writing—review & editing. **Tomoko Fujii**: Conceptualization; Formal analysis; Methodology; Writing—original draft.

## CONFLICT OF INTEREST STATEMENT

The authors declare no conflicts of interest.

## TRANSPARENCY STATEMENT

The lead author Tatsuhiko Abe affirms that this manuscript is an honest, accurate, and transparent account of the study being reported; that no important aspects of the study have been omitted; and that any discrepancies from the study as planned (and, if relevant, registered) have been explained.

## Supporting information

Supporting information.

## Data Availability

The data that support the findings of this study are available on request from the corresponding author. The data are not publicly available due to privacy or ethical restrictions.

## References

[1] Ferreyro BL , Angriman F , Munshi L , et al. Association of noninvasive oxygenation strategies with all‐cause mortality in adults with acute hypoxemic respiratory failure: a systematic review and meta‐analysis. JAMA. 2020;324(1):57‐67. 32496521 10.1001/jama.2020.9524 PMC7273316

[2] Azoulay E , Lemiale V , Mokart D , et al. Effect of high‐flow nasal oxygen vs standard oxygen on 28‐Day mortality in immunocompromised patients with acute respiratory failure: the HIGH randomized clinical trial. JAMA. 2018;320(20):2099‐2107. 30357270 10.1001/jama.2018.14282 PMC6583581

[3] Lemiale V , Mokart D , Resche‐Rigon M , et al. Effect of noninvasive ventilation vs oxygen therapy on mortality among immunocompromised patients with acute respiratory failure: a randomized clinical trial. JAMA. 2015;314(16):1711‐1719. 26444879 10.1001/jama.2015.12402

[4] Monro‐Somerville T , Sim M , Ruddy J , Vilas M , Gillies MA . The effect of high‐flow nasal cannula oxygen therapy on mortality and intubation rate in acute respiratory failure: a systematic review and Meta‐Analysis. Crit Care Med. 2017;45(4):e449‐e456. 27611978 10.1097/CCM.0000000000002091

[5] Rochwerg B , Brochard L , Elliott MW , et al. Official ERS/ATS clinical practice guidelines: noninvasive ventilation for acute respiratory failure. Eur Respir J. 2017;50(2):1602426. 28860265 10.1183/13993003.02426-2016

[6] Abe T , Takagi T , Fujii T . Update on the management of acute respiratory failure using non‐invasive ventilation and pulse oximetry. Crit Care. 2023;27(1):92. 36941729 10.1186/s13054-023-04370-4 PMC10027581

[7] Kang BJ , Koh Y , Lim CM , et al. Failure of high‐flow nasal cannula therapy may delay intubation and increase mortality. Intensive Care Med. 2015;41(4):623‐632. 25691263 10.1007/s00134-015-3693-5

[8] Kangelaris KN , Ware LB , Wang CY , et al. Timing of intubation and clinical outcomes in adults with acute respiratory distress syndrome. Crit Care Med. 2016;44(1):120‐129. 26474112 10.1097/CCM.0000000000001359 PMC4774861

[9] Committee. BTSSoC . Non‐invasive ventilation in acute respiratory failure. Thorax. 2002;57(3):192‐211. 11867822 10.1136/thorax.57.3.192 PMC1746282

[10] Roca O , Messika J , Caralt B , et al. Predicting success of high‐flow nasal cannula in pneumonia patients with hypoxemic respiratory failure: the utility of the ROX index. J Crit Care. 2016;35:200‐205. 27481760 10.1016/j.jcrc.2016.05.022

[11] Nishikimi M , Nishida K , Shindo Y , et al. Failure of non‐invasive respiratory support after 6 hours from initiation is associated with ICU mortality. PLoS One. 2021;16(4):e0251030. 33930089 10.1371/journal.pone.0251030 PMC8087003

[12] Frat JP , Thille AW , Mercat A , et al. High‐flow oxygen through nasal cannula in acute hypoxemic respiratory failure. N Engl J Med. 2015;372(23):2185‐2196. 25981908 10.1056/NEJMoa1503326

[13] He H , Sun B , Liang L , et al. A multicenter RCT of noninvasive ventilation in pneumonia‐induced early mild acute respiratory distress syndrome. Crit Care. 2019;23(1):300. 31484582 10.1186/s13054-019-2575-6 PMC6727327

[14] Coudroy R , Frat J‐P , Ehrmann S , et al. High‐flow nasal oxygen alone or alternating with non‐invasive ventilation in critically ill immunocompromised patients with acute respiratory failure: a randomised controlled trial. Lancet Respir Med. 2022;10(7):641‐649. 35325620 10.1016/S2213-2600(22)00096-0

[15] Ospina‐Tascón GA , Calderón‐Tapia LE , García AF , et al. Effect of high‐flow oxygen therapy vs conventional oxygen therapy on invasive mechanical ventilation and clinical recovery in patients with severe COVID‐19: a randomized clinical trial. JAMA. 2021;326(21):2161‐2171. 34874419 10.1001/jama.2021.20714 PMC8652598

[16] Perkins GD , Ji C , Connolly BA , et al. Effect of noninvasive respiratory strategies on intubation or mortality among patients with acute hypoxemic respiratory failure and COVID‐19: the RECOVERY‐RS randomized clinical trial. JAMA. 2022;327(6):546‐558. 35072713 10.1001/jama.2022.0028 PMC8787685

[17] Yoshida T , Uchiyama A , Matsuura N , Mashimo T , Fujino Y . The comparison of spontaneous breathing and muscle paralysis in two different severities of experimental lung injury. Crit Care Med. 2013;41(2):536‐545. 23263584 10.1097/CCM.0b013e3182711972

[18] Cruces P , Retamal J , Hurtado DE , et al. A physiological approach to understand the role of respiratory effort in the progression of lung injury in SARS‐CoV‐2 infection. Crit Care. 2020;24(1):494. 32778136 10.1186/s13054-020-03197-7 PMC7416996

