# Supplementary material for: Optimal timing for intubation in patients on non‐invasive ventilation: A retrospective cohort study
Source: Health Sci Rep. 2023 Dec 11;6(12):e1757. doi: 10.1002/hsr2.1757 (PMC10713869; doi:10.1002/hsr2.1757)
Supplement: Supplementary file 1 — Supporting information. [file HSR2-6-e1757-s001.docx]

**Supplementary Figure 1. Patient flowchart**

**Supplementary Figure 2. Additional analysis of the relationship between invasive ventilator-free days at 28-day and the number of days from NIV initiation to tracheal intubation including patients who died within 24 hours of non-invasive ventilation**


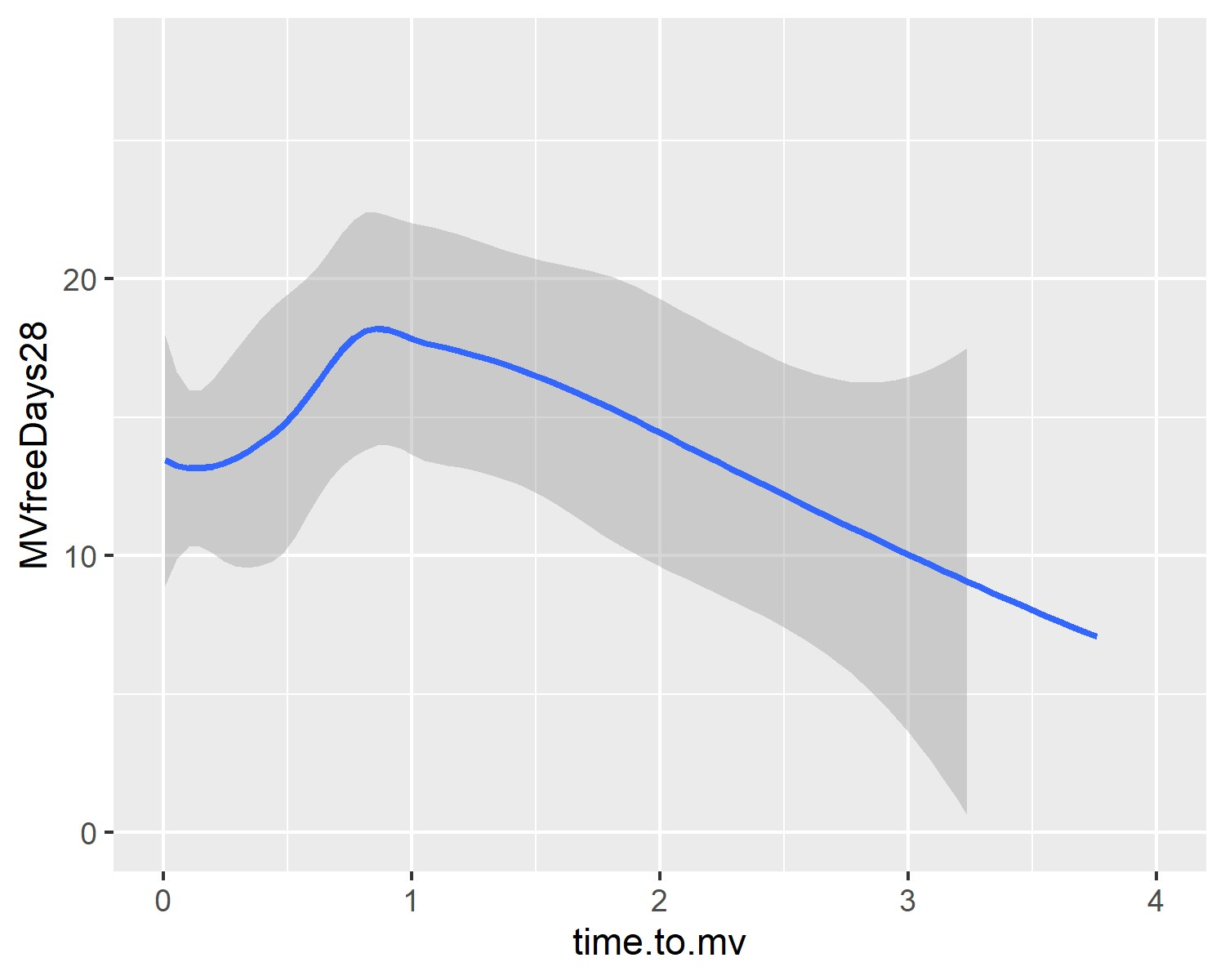


**Supplementary Table 1. Sensitivity analysis of the association between late intubation and 28-day ventilator free days.**

|  | mean difference | 95% confidence interval | p value |
| --- | --- | --- | --- |
| NIV duration of more than 24 hours | -2.665 | [-4.63, -0.70] | 0.008 |
| Δ ROX index | 1.280 | [0.32, 2.24] | 0.009 |
| NIV duration of more than 24 hours | -2.837 | [-4.80, -0.87] | 0.005 |
| Δ P/F ratio | 0.001 | [0.00, 0.00] | 0.007 |
| Δ Respiratory rate | 0.007 | [0.00, 0.01] | 0.003 |
| NIV duration of more than 24 hours | -2.738 | [-0.47, -0.77] | 0.006 |
| ROX index at pre-NIV | -0.074 | [-1.38, 1.24] | 0.912 |
| NIV duration of more than 24 hours | -2.748 | [-4.71, -0.78] | 0.006 |
| P/F ratio at pre-NIV | 0.000 | [0.00, 0.00] | 0.661 |
| Respiratory rate at pre-NIV | -0.001 | [-0.01, 0.00] | 0.667 |
| NIV duration of more than 24 hours | -2.765 | [-4.73, -0.80] | 0.006 |
| ROX index at pre-intubation | 1.157 | [0.23, 2.08] | 0.014 |
| NIV duration of more than 24 hours | -2.807 | [-4.77, -0.84] | 0.005 |
| P/F ratio at pre-intubation | 0.001 | [0.00, 0.00] | 0.004 |
| Respiratory rate at pre-intubation | -0.002 | [-0.01, 0.00] | 0.397 |
